# Superparamagnetic Iron Oxide-Labeled *Leishmania major* Can Be Traced in Fibroblasts

**DOI:** 10.1155/2023/7628912

**Published:** 2023-01-04

**Authors:** Narjes Yektaeian, Shahrokh Zare, Amir Hosein Radfar, Gholamreza Hatam

**Affiliations:** ^1^Department of Parasitology and Mycology, School of Medicine, Shiraz University of Medical Sciences, Shiraz, Iran; ^2^Stem Cells Technology Research Center, Shiraz University of Medical Sciences, Shiraz, Iran; ^3^Basic Sciences in Infectious Diseases Research Center, Shiraz University of Medical Sciences, Shiraz, Iran

## Abstract

**Introduction:**

Leishmaniasis is still a neglected tropical disease that can endanger more than 350 million people among 98 countries. *Leishmania* can survive in fibroblasts as latent inactive forms. This study was conducted to evaluate the role of superparamagnetic iron oxide nanoparticles (SPIONs) in cell culture for tracking the labeled *Leishmania major* in fibroblasts.

**Methods:**

Dextran-coated SPIONs were used for labeling *L. major* in co-culture of fibroblasts with the parasite. To quantify and trace SPION-labeled *Leishmania*, Prussian blue staining was undertaken. Fibroblast characterization was undertaken by real time polymerase chain reaction. Transmission electron microscope (TEM) was used for confirming the entry of the labeled *L. major* to the cytoplasm and the nucleus of the fibroblast.

**Results:**

Fibroblasts were spindle-shaped and adherent to culture flasks. Promastigotes were with thin elongated lance-like morphology with an anterior kinetoplast and an emergent free flagellum. Prussian blue staining revealed that internalized SPIONs were localized within cytoplasm and nucleus of the fibroblasts after 24 hours of culture. Prussian blue staining successfully showed the presence of iron (stained blue) in labeled *L. major* within the fibroblasts. This finding was confirmed by TEM, and labeled *L. major* was detected in the fibroblast cytoplasm and nucleus too.

**Conclusion:**

We can conclude that SPIONs are safe, inexpensive, easy to use, and accurate, and a fast method to label *Leishmania* parasite in cells that the parasite can be latent, such as fibroblasts. These findings can open a new window in diagnosis, pathogenesis, and treatment of cutaneous leishmaniasis and can be added to the literature.

## 1. Introduction

Worldwide, leishmaniasis is a neglected tropical disease that can endanger about 350 million people in at least 72 developing countries and 13 in developed ones [1]. Different forms of leishmaniasis have been reported categorized as (i) cutaneous leishmaniasis (CL) that is a disabling disease when lesions are present in a multiple form; (ii) visceral leishmaniasis (VL) that usually is a fatal form, when left untreated; (iii) muco-cutaneous leishmaniasis (MCL) that is considered as a mutilating disease; and (iv) diffuse cutaneous leishmaniasis (DCL) that is a long-lasting disease, when a deficient cellular-mediated immune response happens [2].

Twenty *Leishmania* species were reported pathogenic for man and 30 sand-fly species as vectors [3]. VL has been reported from 65 countries with annual 500,000 new cases, whereas for CL, annually, 1–1.5 million new cases were demonstrated [4]. More than 90% of CL cases were shown in Saudi Arabia, Algeria, Afghanistan, Iran, Iraq, Syria, Brazil, and Sudan [5, 6]. In CL, an erythematous papule is first observed at the site of inoculation that may enlarge and breaks leading to a painless ulcer. The incubation period is 1–4 weeks that may resolve after 3 months to 2 years [2].

CL rarely causes morbidity, but treatment of the lesions is not easy and usually leaves deep scars on the face or other body surfaces. In Iran, *Leishmania major* enrolls most of the cases of human CL and is classified as acute (less than 12 months), chronic (12 months or longer), and recidivans form [7]. In CL, various species of the genus *Leishmania* were found to infect macrophages of mammalian tissues and enter the dermis through inoculation during the bite of the sand fly vector as promastigotes [7].

These promastigotes have interaction with cellular targets of dermal dendritic cells, neutrophils, macrophages, and eosinophils [7], and when phagocytosed by skin macrophages, they can change into amastigote forms that are released by exploding the cells. The parasite can further infect reticuloendothelial system, such as the bone marrow, liver, and spleen [8]. Recently, it has been demonstrated that parasites enter other cell types, such as fibroblasts, amniotic epithelial cells, kidney cells, neutrophils, eosinophils, and dendritic cells as latent inactive forms [9, 10].

Within fibroblasts, it was shown that the parasites can persist for 3–7 days, with a replication time of 18–20 days [11]. It was found that human fibroblast can uptake *Leishmania amazonensis* amastigote for periods of six or more hours and reach a peak of 60% on days 2 or 3 and then decrease to zero. For promastigotes of *Leishmania braziliensis*, the same type of infection course was noticed, whereas promastigotes of *Leishmania donovani* can easily enter human skin fibroblasts [11]. These findings reveal the evolution of the *Leishmania* infection in fibroblasts and macrophages and the key role in escape mechanisms developed by the parasites.

The diagnosis is mostly upon clinical and epidemiological aspects of disease, parasite detection (stained smears, culture, and histology), immunological techniques, such as Montenegro skin test, immunofluorescence assay, enzyme-linked immunosorbent assay, and molecular devices, such as isoenzyme characterization and polymerase chain reaction (PCR) [12, 13]. Superparamagnetic iron oxide nanoparticles (SPIONs) have been widely used in cell labeling of various cells using magnetic resonance imaging and Prussian blue staining to detect and track the labeled cells [14]. SPIONs are very small synthetic *γ*-Fe_2_O_3_ (maghemite) or Fe_3_O_4_ (magnetite) particles, coated with certain biocompatible polymers, such as dextran or polyethylene glycol, to facilitate their conjugation and easy transport in blood [15]. Several attempts have been made to correlate transmission electron microscopic (TEM) changes with diagnosis of human leishmaniasis [14–16], but none of them has assessed fibroblasts infected with SPIONs labeled *Leishmania*. This study was undertaken to determine the role of SPIONs in *in vitro* detection and tracing of labeled *L. major* in fibroblast cells.

## 2. Materials and Methods

### 2.1. Fibroblast Cultivation and Characterization

Human foreskin was used for isolation of fibroblasts. The tissue samples were first washed three times in phosphate buffer saline (PBS: Sigma–Aldrich, USA), penicillin, streptomycin, and amphotericin B (100 U/ml, 100 *μ*g/ml, and 0.25 *μ*g/ml, respectively; Invitrogen, USA), and the adipose tissues, blood vessels, and debris were removed. The remaining was cut into small pieces, and then, 0.5% dispase (Gibco, USA) was added to the chopped sample at 4°C for 18 hours. The epidermal layer was later separated, and the remained dermal layer was cut more into 1–2 mm^3^ pieces, whereas 0.1% collagenase type 1 (Thermo Fisher Scientific, USA) was later added at 37°C for 4 hours.

The enzymatic reaction was stopped by adding culture medium including Dulbecco's Modified Eagle's Medium/F12 (DMEM/F12, Invitrogen) with 10% fetal bovine serum (FBS: Gibco, USA), and the product was ultimately passed through a 70 *μ*m cell strainer, and ultimately centrifuged and suspended in DMEM F12, 10% FBS, and 1% penicillin/streptomycin. The precipitated fibroblast cells were seeded in tissue culture flasks and transferred into incubator at 37°C, 5% CO_2_ and saturated humidity while the medium was changed every 3 days until 80% confluence. Fibroblasts were then trypsinized, using 0.025% 1× trypsin–EDTA (Invitrogen, USA), for further sub culturing. To determine the cell viability, trypan blue staining was used. Phenotypic characterization of fibroblasts was conducted by assessment of morphology to be spindle-shaped and by real time PCR for fibroblasts special markers including matrix metalloproteinase-1 (MMP1), matrix metalloproteinase-3 (MMP3), CD10, and CD26 and negative control CD106 and integrin alpha 11.

### 2.2. *L. major* Cultivation

The reference strain of *L. major* (MRHO/IR/75/ER) was provided from Medical School, Shiraz University of Medical Sciences, Shiraz, Iran. The parasite was cultivated in RPMI-1640 containing 10% FBS, 100 IU/mL penicillin, and 100 *μ*g/mL streptomycin. The cultures were incubated at 25°C, and the stationary phase of parasite was obtained after 6 days. For co-culturing the fibroblast cells and parasites, the fibroblast cells were removed from culture flask as described, centrifuged at 13,000*g* for 3 minutes, washed three times with PBS pH: 7.2, and seeded in 24-well plates. It permitted to adhere (6 hours) to the bottom of the wells in order to co-culture with the *L. major*. One week later, the culture medium was drained, and new culture medium was added instead. Within 2–4 days, fibroblasts were grown in the wells.

*L. major* promastigotes were centrifuged for 3 minutes at 3000*g*, counted, and were added to fibroblast wells in a ratio of 1 : 10 parasites, followed by incubation at 37°C in a 5% CO_2_ for 4–14 hours. After that, culture medium was washed to remove non-phagocytized parasites. After 12 hours, the culture was washed three times with PBS buffer to eliminate the remained parasites.

### 2.3. Labeling of *L. major*

To label the parasites, dextran-coated SPIONs (micromod Partikeltechnologie, GmbH, Rostock, Germany) were used to track the parasite. The mean hydrodynamic particle diameter may vary in the range of 20–100 nm, and they are preferably applied for detection and tracing purposes [17, 18]. The nanoparticles used in our study were about 50–60 nm in size with an unmodified dextran surface (Nanomag^®^-D-spio nanoparticles, Product code of 79-00-201) and could be stored at 2°C–8°C. In summary, *Leishmania* was transferred in DMEM for 4 hours in the absence of FBS and penicillin and streptomycin. Media was later changed to DMEMF12 containing 1 *μ*L/ml Lipofectamine and the same volume containing 90 *μ*g/ml of SPIONs left in room temperature for 15 minutes and later incubated for 20 hours at 37°C. The parasite was washed three times with PBS, and the media containing Lipofectamine and iron oxide was removed.

To quantify the parasite uptake, *L. major* labeled with SPIONs was fixed in 10% formalin and then was treated in a solution of 1 : 1 ratio of 20% aqueous HCl and 10% aqueous potassium ferrocyanide as working solution for 20 minutes in room temperature. Cells were later washed three times in distilled water and counterstained with nuclear fast red for 5 minutes, rinsed twice in distilled water, and permeabilized using 95% methanol for 15 minutes at room temperature. Finally, the presence of iron was visualized using Prussian blue staining shown as bright blue for iron oxide, red color for nuclei, and looking pink for the cytoplasm. A light microscope (FSX100, Olympus, Japan) was used for cell imaging.

### 2.4. Transmission Electron Microscopy

*In vitro* fibroblast cells infected to *L. major* labeled with SPIONs were fixed in 3% cold glutaraldehyde, buffered with 0.2 M sodium cacodylate, and fixed again in 1% osmium tetroxide. They were further dehydrated through ascending series of ethanol and were embedded in agar-100 resin to provide semithin sections (1 *μ*m in thickness). The sections were cut by ultramicrotome and stained with toluidine blue and were assessed under a light microscope to confirm a proper preparation and orientation. Ultrathin sections were cut from selected regions, mounted on copper grids, double stained with uranyl acetate and lead citrate, and were finally evaluated using a TEM and screened for presence of SPIONs in the labeled parasite.

### 2.5. Statistical Analysis

Statistical analysis method was undertaken using the SPSS software (version 2, Chicago, IL, USA), and *t*-test was used for comparison. GraphPad was used to show the figures.

## 3. Results

### 3.1. Characterization of Fibroblast Cells

**Figure 1:**
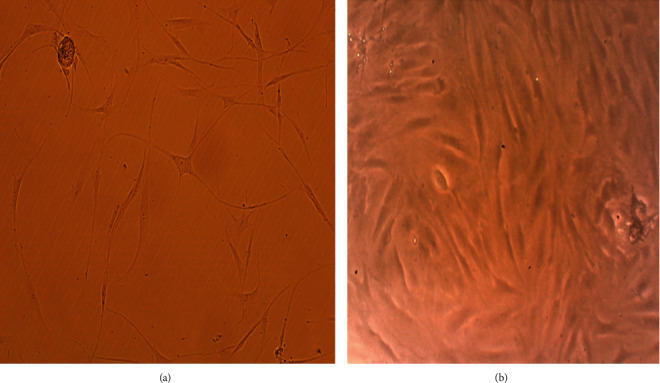
Fibroblast spindle shape morphology on day 4 [(a) ×40 and (b) ×200].

**Figure 2:**
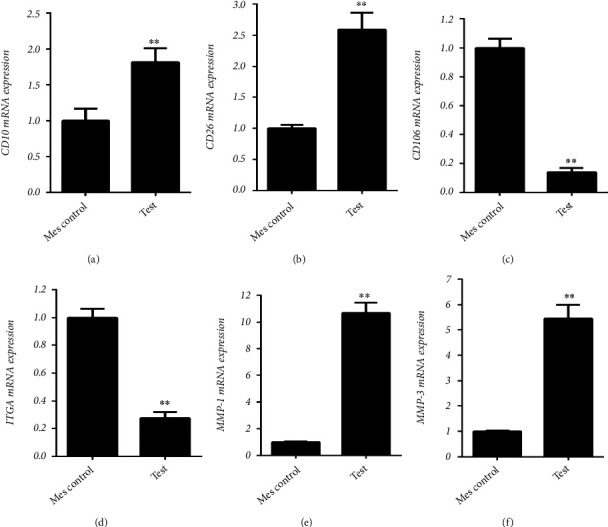
Human fibroblasts were positive for expression of (a) CD10, (b) CD26, (c) CD106, (d) integrin alpha 11 (ITGA11), (e) matrix metalloproteinase (MMP) 1, and (f) MMP3 (∗*p* ≤ 0.05 and ∗∗*p* ≤ 0.01) [10].

Phenotypic characterization of fibroblasts was conducted by assessment of morphology to be spindle-shaped and adherent to culture flasks (Figure 1). With real time PCR, human fibroblasts were positive at the expression of CD10, CD26, MMP-1, and MMP-3, and negative for CD106 and integrin alpha 11 (Figure 2).

### 3.2. *L. major* Morphology

**Figure 3:**
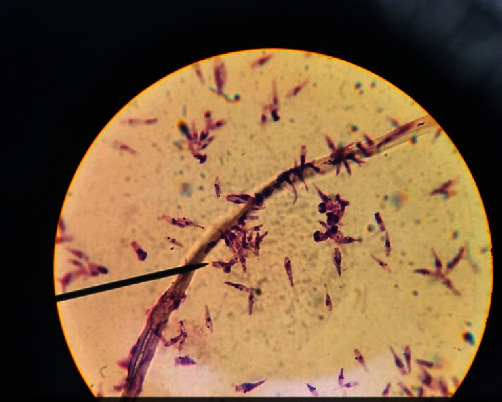
Promastigotes were thin elongated lance-like in shape on fourth day of culture (×100).

**Figure 4:**
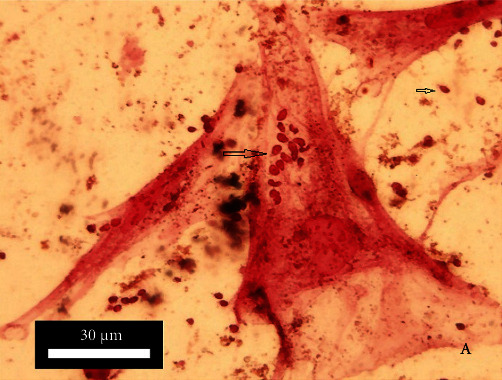
Parasite stained by SPIONs (iron nanoparticles) 3 days after exposure to the fibroblast. The promastigotes differentiate according to its elongated shape and size, and spread in both of nucleus and cytoplasm of fibroblast. Magnification 200×.

Promastigotes were with thin elongated lance-like morphology with an anterior kinetoplast and an emergent free flagellum (Figure 3). *L*. *major* amastigotes were detected 24 hours after co-culturing with fibroblasts. Parasites labeled by SPIONs and stained by Prussian blue were demonstrated in blue color (Figure 4).

### 3.3. *L. major* Labeling

Prussian blue staining revealed that internalized SPIONs were localized within cytoplasm and nucleus (Figure 4) of the fibroblasts after 24 hours culture. Prussian blue staining successfully showed the presence of iron (stained blue) in labeled *L. major* within the fibroblasts.

### 3.4. Transmission Electron Microscopy

**Figure 5:**
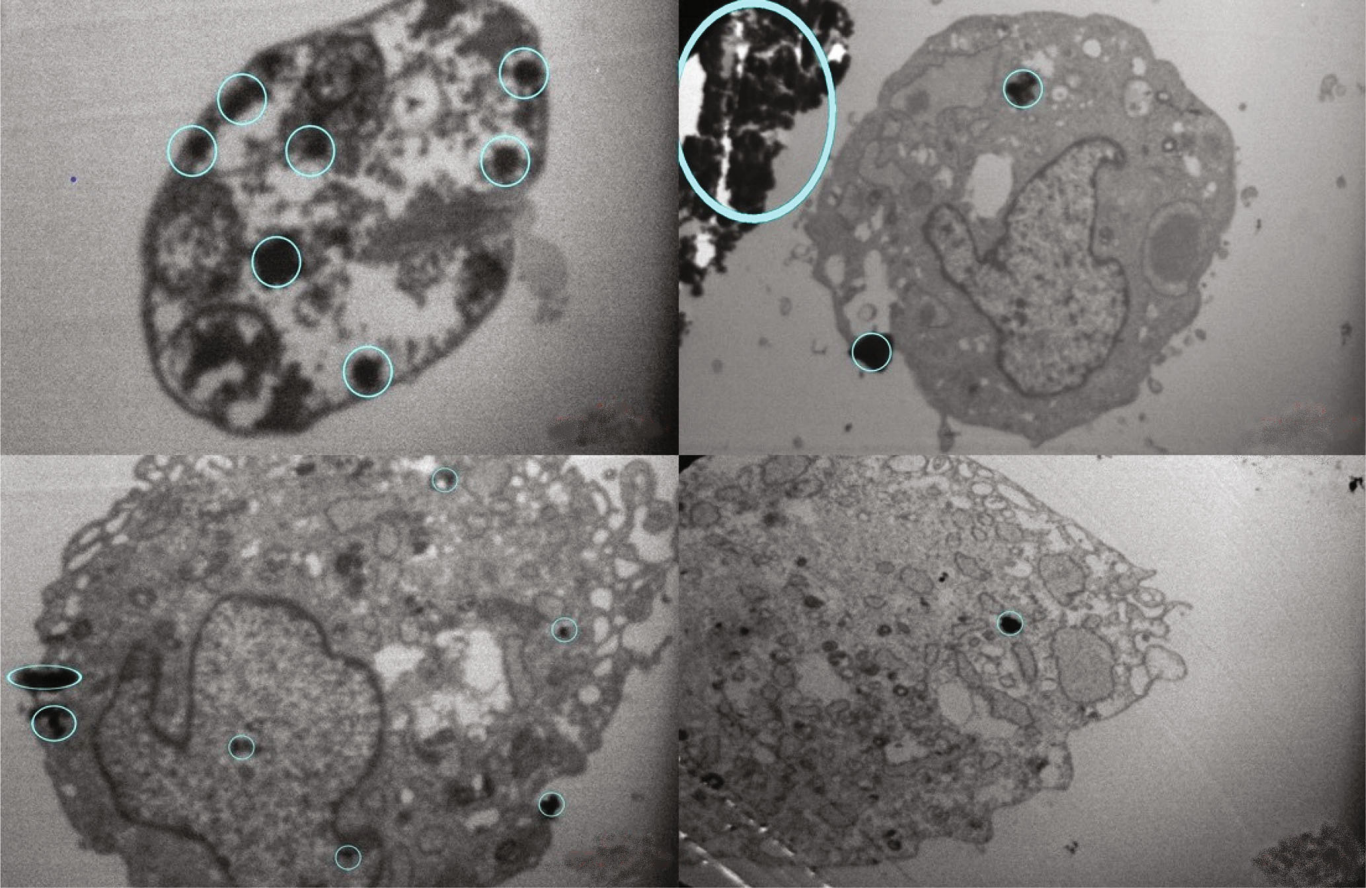
TEM micrograph of fibroblast cells infected to *L. major* labeled with SPIONs denoting to the presence of iron oxide nanoparticles in the cells encircled in blue color.

TEM micrograph of fibroblast cells infected to *L. major* labeled with SPIONs was shown in Figure 5 denoting to numerous labeled *Leishman* bodies in the cells. The presence of iron oxide nanoparticles in fibroblast cells was encircled in blue color.

## 4. Discussion

The *in vitro* infection of fibroblasts cells with *Leishmania* species, such as *L. donovani*, was previously established and was shown to survive within the cells for 3–7 days [10]. Identically, we were also successful to infect the fibroblasts with *L. major*. Detection of parasite in fibroblast has been undertaken using different methods, such as special surface antibody marking, immunoperoxidase staining, laser scanning confocal microscopy, and TEM, but they are considered as expensive and time consuming methods, and need expert persons. To visualize and trace the labeled cells, SPIONs have been used as a fast, easy, and cost-effective method that is visualized by Prussian blue staining [16]. SPIONs have been used for labeling of various cell types both *in vitro* and *in vivo* [19]. They have been used to trace stem cell transplantation in a target organ. Bulte et al. successfully used SPION-labeled mesenchymal stem cells (MSCs) for tracking in Human HeLa and GLC-28 cells, rat CG-4 cells, and mouse 3T3 and C2C12 cells all showed a comparable degree of uptake [20]. Moore et al. used SPIONs for labeling of MSCs and could trace the cells with no side effect on their morphology or viability [21]. In the presence of SPIONs, the tracing of immune responsive cells has been undertaken increasing the chance for diagnosis and monitoring of the treatment without any impairment [22]. In our study, labeling of parasite with SPIONs has been conducted with good results that could detect and track labeled *L. major* after staining with Prussian blue. The labeling and staining could easily visualize, detect, and track *L. major* within the fibroblasts denoting to successful conjugation and transportation of the SPIONs [15, 18]. The chance for detection and tracking of SPIONs labeled cells was previously demonstrated in labeling of neural stem cells [17], neurons [18], progenitors [23], Schwann cells, olfactory ensheating cells [19], transferring receptors [24], Sendai viral envelopes [20], and human immunodeficiency virus (HIV)-Tat peptides [25]. In addition, SPIONs were also used for direct detection of solid tumors, because SPIONs are passively accumulated at the tumor site due to the presence of a leaky vasculature as well as macrophage uptakes [21]. SPIONs have been applied in central nervous system for imaging of strokes [26], multiple sclerosis [24], brain tumors [25], and carotid atherosclerotic plaques [27] exhibiting the important role of SPIONs in cell labeling and further tracking of these nanoparticles similar to our findings. Clean delineation of metastases in the brain was also possible through SPIONs use, approximately 24 hours following the use of these nanoparticles clarifying the prominent role of nanoparticles for further visualization and cell tracing [28]. The potential of *in vivo* cell tracing was shown in SPION-loaded T cells too [22]. Several researchers reported labeling of bacteria using iron oxide nanoparticles. Shemisa et al. successfully traced iron particles in mycobacterial infections using Prussian blue staining [29]. Iron oxide particles were used in Baculo viruses for visualization and tracking of the viruses [30]. Hoerr et al. labeled *Staphylococcus aureus* colonies with iron oxide and could further track the bacteria [31]. Similarly, in line with above mentioned studies, we visualized the fibroblast with *Leishmania* labeled SPIONs after staining with Prussian blue, whereas SPIONs did not have any side effect for cell adhesion behavior or morphology [32]. Identical findings were reported for dextran-coated SPIONs labeling the Tat-internalizing peptides with no significant side effects on cell viability, clonogenic efficiency, immunophenotypic changes, or biodistribution in human hematopoietic stem cells [33]. Our results also revealed no side effects and changes in cell viability and efficiency that are in line with previous studies. Based on histological and electron microscopy of CL, the parasite was shown in lymphocytes, plasmocytes, macrophages, and skeletal muscles [34]. In electron microscopy of *L. donovani*, it was demonstrated that the parasite was visible with double membrane, nucleus, and basal body [22].

## 5. Conclusion

We can conclude that SPIONs are safe, inexpensive, accurate, and a fast method to label *Leishmania* parasites in cells that the parasite can be latent inside, such as fibroblasts. These findings can open a new window in detection, tracking, diagnosis, pathogenesis, and treatment of CL, and can be added to the literature.

## Data Availability

The data used to support the findings of this study are included within the article.
